# EBSD Analysis of Metal Matrix Nanocomposite Microstructure Produced by Powder Metallurgy

**DOI:** 10.3390/nano9060878

**Published:** 2019-06-12

**Authors:** Íris Carneiro, Filomena Viana, Manuel F. Vieira, José V. Fernandes, Sónia Simões

**Affiliations:** 1 CEMMPRE, Department of Metallurgical and Materials Engineering, University of Porto, R. Dr. Roberto Frias, 4200-465 Porto, Portugal; up201207199@fe.up.pt (Í.C.); fviana@fe.up.pt (F.V.); mvieira@fe.up.pt (M.F.V.); 2 INEGI—Institute of Science and Innovation in Mechanical and Industrial Engineering, R. Dr. Roberto Frias, 4200-465 Porto, Portugal; 3 CEMMPRE, Department of Mechanical Engineering, University of Coimbra, Rua Luís Reis Santos, Pinhal de Marrocos, 3030-788 Coimbra, Portugal; valdemar.fernandes@dem.uc.pt

**Keywords:** metal matrix nanocomposites, carbon nanotubes, electron backscattered diffraction, grain boundary, dislocation structure, recrystallization

## Abstract

The development of metal nanocomposites reinforced by carbon nanotubes (CNTs) remains a focus of the scientific community due to the growing need to produce lightweight advanced materials with unique mechanical properties. However, for the successful production of these nanocomposites, there is a need to consolidate knowledge about how reinforcement influences the matrix microstructure and which are the strengthening mechanisms promoting the best properties. In this context, this investigation focuses on the study of the reinforcement effect on the microstructure of an Ni-CNT nanocomposites produced by powder metallurgy. The microstructural evolution was analysed by electron backscattered diffraction (EBSD). The EBSD results revealed that the dispersion/mixing and pressing processes induce plastic deformation in the as-received powders. The dislocation structures produced in those initial steps are partially eliminated in the sintering process due to the activation of recovery and recrystallization mechanisms. However, the presence of CNTs in the matrix has a significant effect on the dislocation annihilation, thus reducing the recovery of the dislocation structures.

## 1. Introduction

The growing interest in the development of metallic matrix nanocomposites (MMNCs) is attributed to the extraordinary mechanical, physical and chemical properties that can be obtained by combining different types of materials. This development depends on several factors, since the improvement in the mechanical properties of the metallic matrices is strongly related, not only to the composition of the reinforcement material used, but also to its size, morphology, volume fraction, interfacial bonding to the matrix and the uniformity of its dispersion in the metal matrix [1,2].

MMNCs are good candidates for wide use in components for various sectors, such as the automotive and aerospace industries [3,4]. Ni [3,4,5,6], Cu [7,8,9,10] and Al [11,12,13,14] represent the materials most commonly studied as matrices of these nanocomposites, due to the nanoreinforcements’ strengthening potential, thus becoming advantageous alternatives to conventional materials. Among the various nanoreinforcements investigated, carbon nanotubes (CNTs) have aroused great interest amongst the scientific community [15,16].

MMNCs reinforced with CNTs can be produced using powder metallurgy routes [5,6,10,17,18,19]. However, the expected improvement in properties was not fully achieved, due to several factors. The homogeneous dispersion of the CNTs is one of the main challenges in the production of nanocomposites, since they tend to agglomerate [1]. Ball-milling [20,21] and ultrasonication [17,22,23] are some of the most effective methods used in promoting the dispersion of the CNTs and in the production of MMNCs. The efficiency of these dispersion techniques depends on some parameters, such as time, energy and liquids used (ethanol, isopropanol, ethylene glycol or acids), which need to be carefully selected in order to obtain a uniform dispersion without a significant CNT damage. Understanding and identifying the nanocomposite strengthening mechanisms are also crucial. Load transfer from matrix to reinforcement [9,24,25] is the most widely reported strengthening mechanism, but others have been identified in MMNCs [19,20,21,22,23,24,25,26]. Grain refinement, as well as the Orowan hardening [9,26,27,28,29,30], or even solid solution strengthening by carbon atoms that diffuse from the reinforcement to the matrix [31], are some of the possible mechanisms. Particle strengthening, induced by a second phase formed by the reaction of the reinforcement with the metal matrix, has been reported [26,27,32,33]. Finally, strain hardening (the increase in dislocation density) has also been identified as an important strengthening mechanism of MMNCs [34,35].

Although several authors report the effect of processing conditions, amount, dispersion and surface treatment of CNTs on the mechanical properties of nanocomposites [12,18,19,36,37,38], there are still only a few studies [3,39] that focus on the microstructural changes during the production process. Among the changes in the MMNCs that can be attributed to the presence of CNTs, the grain size of the matrix and/or texture formation should be emphasized. Suárez et al. [3] characterized the grain size of Ni-CNT nanocomposites, with 1, 2, 3 and 5 wt.% of CNTs, produced by powder metallurgy. The characterization was performed by scanning electron microscopy (SEM) and electron backscatter diffraction (EBSD) and showed an increase in pores and clusters with the CNT content. After sintering, Ni samples showed larger grain sizes than those of nanocomposites, confirming that the presence of CNTs influences the grain growth of the matrix during sintering [39]. In our previous study [5], the presence of CNTs in the Ni matrix also induced microstructural changes in the nanocomposite during the powder metallurgy process. The nanocomposites, unlike the samples without CNTs, exhibited a dense dislocation structure, whereas the pure Ni consisted mainly of recrystallized grains with their associated low dislocation density.

Microstructural changes promoted by the presence of the reinforcement during the production process are still a little-understood phenomenon. In this context, the main objective of this work is to study the effect of CNTs during the powder metallurgy process on the final microstructure of the nanocomposites. The EBSD analysis was used, this being a powerful technique to obtain several microstructural characteristics, such as the size and shape of the grains, the crystallographic orientation and the evidence of the formation of dislocation microstructure.

## 2. Materials and Methods

The nickel powders used in the production of MMNCs (from Goodfellow Cambridge Ltd., Huntingdon, UK), exhibit a purity of 99.5%. The CNTs used (from Fibermax Nanocomposites Ltd., London, UK) are multi-walled (MWCNTs) with an inner and outer diameter of 5 and 19 nm respectively. The as-received MWCNTs are entangled, with a large aspect ratio and a bamboo-type structure characteristic of the production technique (chemical vapour deposition).

The effect of the powder metallurgy process was evaluated through the characterization of the materials resulting from each step (dispersion/mixture, pressing, and sintering). The as-received Ni powders and samples without reinforcement material produced under the same conditions were also analysed for comparison purposes. The selection of the production conditions (such as CNT concentration, dispersion/mixture method and conditions, and sintering temperature) were based on the results of previous works [6,37]. The dispersion and mixture of 1.00 vol.% MWCNTs in Ni powders were performed in one step, by ultrasonication using isopropanol for 15 min using 20,400 kHz, as optimized for Al-CNT nanocomposites [12,15]. The mixtures were then pressed with 900 MPa, and pressureless sintered at 950 °C for 120 min in a vertical furnace under a vacuum of 10^−2^ Pa.

Microstructural characterization was performed by optical microscopy (OM) (DM 4000, Leica Microsystems, Wetzlar, Germany), SEM and EBSD using a high-resolution FEI QUANTA 400 FEG SEM (FEI Company, Hillsboro, OR, USA) equipped with a TSL-EDAX EBSD Unit. The microstructural analysis carried out in OM enables a global characterization of the samples at low magnifications in order to evaluate the size, distribution and morphology of the Ni powders, for instance. This evaluation was performed through the software Leica Application Suite (Leica Microsystems, Wetzlar, Germany) and was assessed by measuring the size of 300 particles and the roundness of 5500 particles. The average grain size of the particles was determined by measuring 100 grains.

The EBSD enables information to be obtained on microstructural characteristics with a small volume of interaction and high resolution. Usually, the reported values of spatial resolution of EBSD are less than 50 nm, although the resolution is dependent on the SEM operation conditions and EBSD detectors and can be influenced by the sample [40,41]. The EBSD analysis was performed considering a minimum step size of 0.03 μm. The software used for EBSD analysis was the TSL OIM Analysis 5.2 (EDAX Inc., Mahwah, NJ, USA).

The grain size and the crystallographic orientation were characterized by inverse pole figure (IPF) maps. The different colours of the IPF maps are related to crystallographic orientations that allow for the determination of grain size, but also for the study of texture formation. Image quality (IQ) maps are associated with the quality of the EBSD patterns, since the brighter areas signify higher pattern quality. These maps are useful for highlighting the microstructural characteristics, since they clearly show the regions without an EBSD sign. A clear example of one of these characteristics is the grain boundaries that are characterized by a weak EBSD pattern due to the overlapping of diffraction patterns from neighbouring grains.

The microstructure of the nanocomposites was also characterized by Kernel average misorientation (KAM) maps and by the evaluation of the grain boundary misorientation. The KAM maps show the average misorientation around a measurement point for a defined set of nearest neighbour points and in this work, it was considered the second neighbour. Each colour of these maps corresponds to a misorientation angle, with a maximum of 15°. To evaluate the type of grain boundary, a crystallographic misorientation between 15° and 180° defined high-angle boundaries and a misorientation between 5° and 15° corresponded to low-angle boundaries. As the metal matrix used in this study was characterized by the presence of twin boundaries, they were also considered and defined as Σ3 and Σ9, with misorientations of 60.0° and 38.9° respectively [42,43].

Grain orientation spread (GOS) maps that show the deviation between the average orientation of the grain and each point within the grain were also performed. These maps are frequently used to investigate the recovery and recrystallization processes [42,44,45,46].

## 3. Results and Discussion

### 3.1. Characterization of As-Received Ni Powders

The characterization of as-received Ni powders is fundamental in following up the microstructural evolution of the nanocomposites during the powder metallurgy steps.

**Figure 1:**
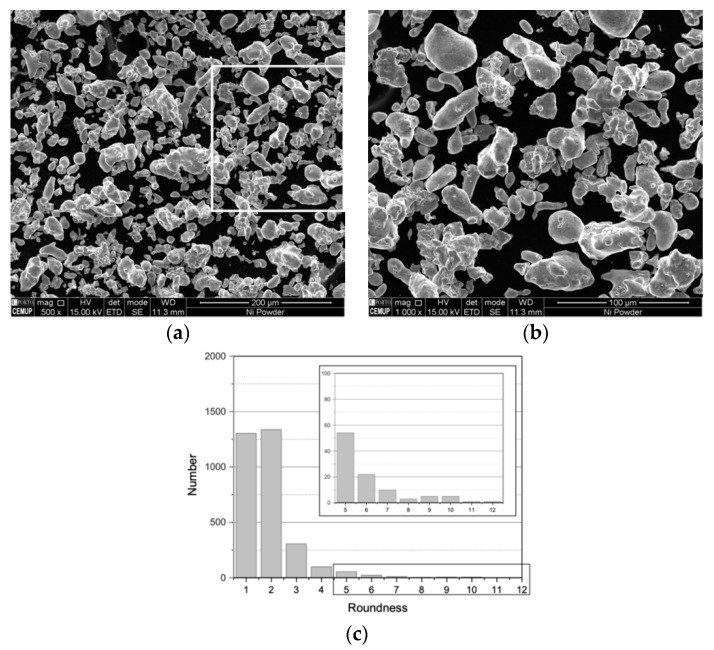
As-received Ni powders: (**a**) and (**b**) scanning electron microscopy (SEM) images and (**c**) roundness distribution.

SEM images of Figure 1 show the morphology of as-received Ni powders. The particle size exhibits a D_50_ of 15 µm, as already mentioned in previous studies [6]. Particles up to 60 µm in size were observed. It is evident that the powders do not have an entirely spherical morphology, which is illustrated in the distribution of roundness shown in Figure 1c. Although most particles present a roundness value close to 1 (1 being the spherical shape), there are still many with high roundness values.

**Figure 2:**
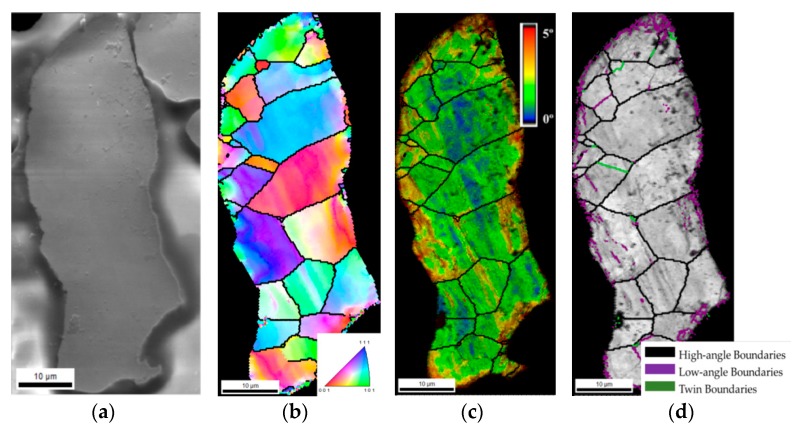
As-received Ni particle: (**a**) SEM image, (**b**) inverse pole figure (IPF), (**c**) Kernel misorientation map (KAM) superimposed image quality (IQ) map, (**d**) grain size map with high-angle, low-angle and twin boundaries.

The microstructure of the as-received Ni powders was characterized by EBSD analysis. Figure 2 shows the SEM image, the IPF map, the KAM map, superimposed with IQ, and grain size map, with different grain boundaries identified by different colours (high-angle, low-angle, and twin boundaries), for an example of Ni particle. The IPF map shows a representative example of an Ni particle. These particles are composed of several grains with a mean grain size of 6.9 ± 4.0 μm. As no preferential orientation of the grains was observed, it could be concluded that the powders have no texture. Based on the results of the KAM maps, it is possible to detect a high misorientation at the surface of the particle. This misorientation is associated with a high density of low-angle boundaries, as observed in the grain size map in Figure 2d. This misorientation can result from the plastic deformation that possibly occurred during production and which has a significant effect on their surfaces.

### 3.2. Microstructural Evolution During Powder Metallurgy Processing

**Figure 3:**
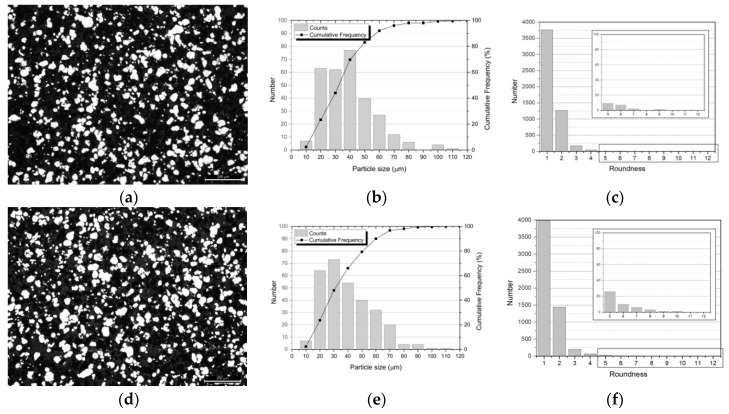
OM image, particle size and roundness distributions of (**a**), (**b**) and (**c**) Ni ultrasonicated powders; (**d**), (**e**) and (**f**) Ni-CNT ultrasonicated powders.

As powder metallurgy involves several steps (mixing, pressing and sintering), the final microstructure is clearly influenced by the changes that occur throughout the process. In the case of the Ni-CNT nanocomposite, the presence of the reinforcing material may cause additional changes. Figure 3 shows the OM images of the powders after the dispersion/mixture technique, as well as the particle size and the roundness distributions, with and without CNTs.

After this processing step, Ni and Ni-CNT particle size distributions exhibited a D_50_ of 33 and 30 µm respectively, and a maximum particle size of 110 µm, showing a significant increase compared to the as-received Ni powders (D_50_ of 15 µm and a maximum particle size of 60 µm). This increase can be associated with the particle agglomeration that occurred during the dispersion/mixture process. Another important aspect to be outlined is that this agglomeration led to an improvement in the powder’s roundness; both samples (Figure 3c,f show a high number of round or nearly round particles compared with the as-received powders (Figure 1c). The presence of CNTs did not induce morphological changes in this processing step, since no significant differences between samples with and without CNTs were detected.

**Figure 4:**
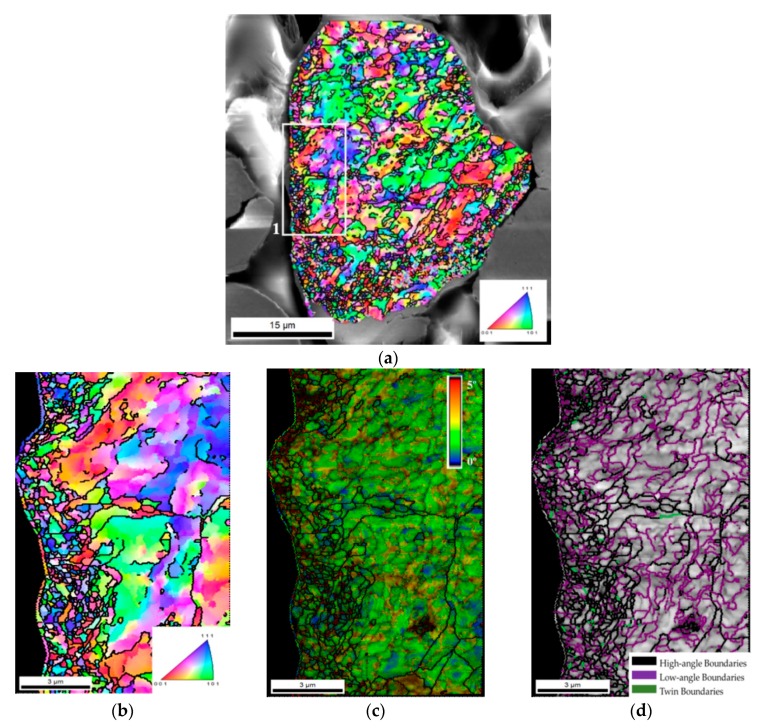
(**a**) SEM image with superimposed IPF map. (**b**), (**c**) and (**d**) IPF map, KAM superimposed on IQ map and grain size map of Region 1 marked in (**a**), respectively.

The influence of the dispersion/mixing process on the microstructure of the powder was evaluated by EBSD. Figure 4 shows the IPF, KAM and grain size maps of an Ni particle after this process. From these results, it is clear that the process induces a significant change in the microstructure of the powder, by reducing the grain size (IPF map). However, no preferred crystallographic orientation was detected, indicating that the process did not lead to texture formation. In contrast to what was observed for the as-received Ni particles, the powders dispersed, and when mixed by the ultrasonication process presented a high density of low-angle grain boundaries, which can be confirmed by the grain size map of Figure 4b. This increase in the density of the grain boundaries is surely associated with plastic deformation occurring during the process. Plastic deformation in nickel is known to promote the rearrangement of dislocations in cells. Plastic deformation increases the dislocation density in cell boundaries, thus increasing misorientation between adjacent cells and leading to the formation of low-angle boundaries. A region on the surface of the particle marked in Figure 4a was observed in more detail through IPF, KAM and grain size maps (Figure 4b–d). These results show that the effect of dispersion and ultrasonication on the formation of new grains is more pronounced at the surface. It is important to note that the process does not act in the same way on all particles, since smaller and rounder particles are less deformed.

**Figure 5:**
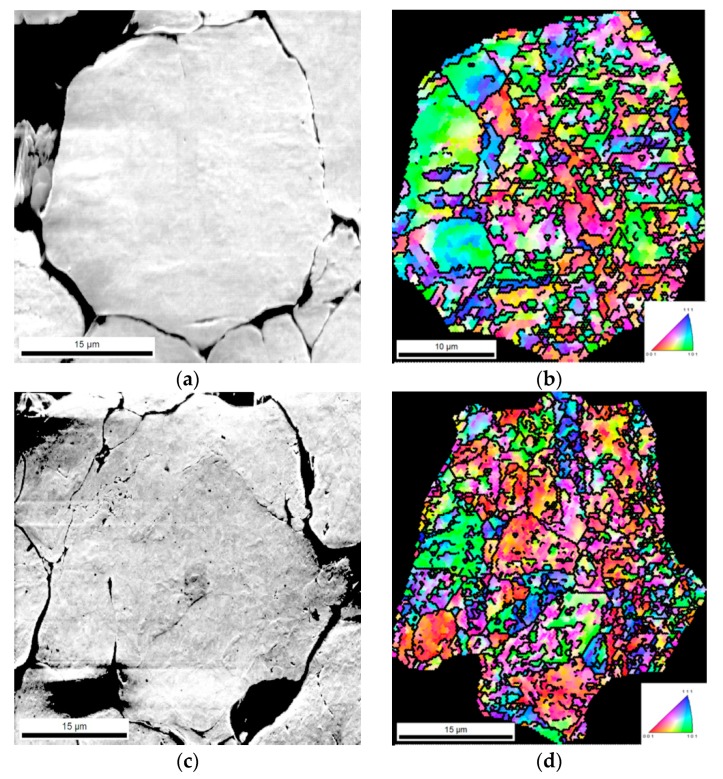
SEM and IPF maps of (**a**), (**b**) Ni powder, (**c**) and (**d**) Ni-CNT powder after pressing.

The SEM images and IPF maps in Figure 5 show examples of Ni and Ni-CNT particles after pressing. The microstructure did not appear to have significantly changed during pressing when compared with the microstructure of the particles after dispersion/mixture (Figure 4). In addition, there are no noticeable differences between Ni and Ni-CNT powder IPF maps, suggesting that the reinforcing material did not greatly influence the microstructural evolution during this stage of production.

Similar results have already been reported in Ni-CNT nanocomposites [5], where these colour gradients were associated with the formation of dislocation cells. These results showed that the presence of CNTs did not induce significant microstructural changes during the dispersion/mixture and pressing processes.

**Figure 6:**
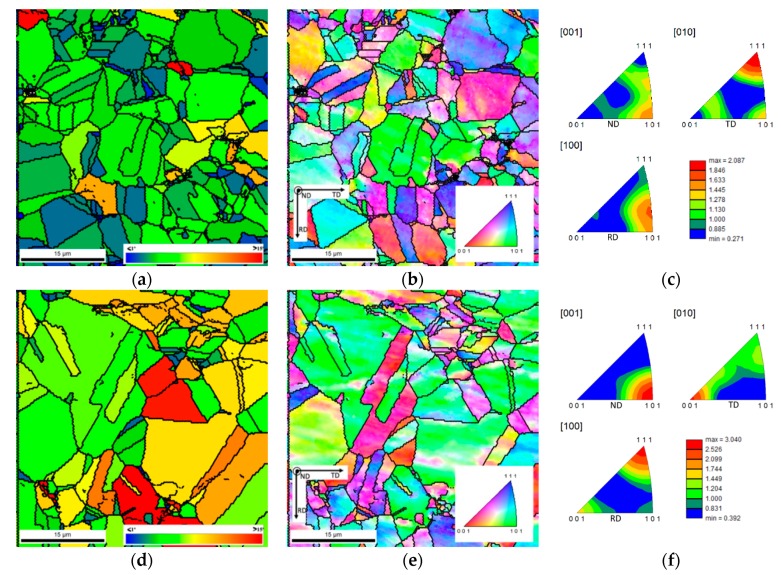
Grain orientation spread (GOS) maps and IPF maps and charts of (**a**), (**b**) and (**c**) sintered Ni, (**d**), (**e**) and (**f**) sintered Ni-CNT nanocomposites.

After sintering, the microstructures of the samples with and without CNTs showed some differences. GOS and IPF maps can be observed in Figure 6. The microstructure of the samples is characterized by the presence of equiaxed grains with some twins. Regarding grain size, both samples had higher average grain size after sintering than that of as-received Ni powders. The mean grain size of both samples was similar; for the Ni sample it was 11.7 ± 7.5 μm, with 37.5 μm as the maximum value, and for the Ni-CNT nanocomposite it was 9.8 ± 6.9 μm, with 26.6 μm as the maximum value.

The slightly smaller average grain size for the nanocomposite is associated with regions of smaller grains observed in the vicinity of CNTs clusters and with the effect of CNTs on recovery, recrystallization and grain growth that occurs during sintering. These smaller grains indicate that CNTs could be acting as obstacles to dislocations, hindering recovery, and leading to dislocation accumulation that favours the appearance of a larger number of recrystallization nuclei in these sites, or as obstacles to the grain boundary movement, thus reducing grain growth kinetics during sintering.

Figure 6 also shows that misorientation inside grains significantly decreased after sintering. This microstructural evolution confirms the occurrence of recrystallization during sintering. Recrystallized grains have low dislocation density. The evaluation of the recrystallization process that occurred during sintering was performed through GOS maps. The recrystallized grains were assumed to be those with an orientation spread of 1° or less (in blue, Figure 6a,d), based on the work of Bair et al. [42]. The results regarding GOS maps revealed that the Ni samples are characterized by a lower orientation spread inside the grains, with green and blue being the predominant colours of these maps, while the Ni-CNT nanocomposites exhibit green and yellow (3 to 7° misorientations) as predominant colours. These results indicate that the recrystallization did not occur to the same extent for both samples. Therefore, it is possible to realise that the recovery and recrystallization of Ni was more effective than in the nanocomposite, which confirms that the CNTs hinder these processes.

Inverse pole figures revealed that the presence of the CNTs induces changes in the crystallographic orientation of the nanocomposites when compared with the sample without reinforcement. The nanocomposites exhibited a more marked texture than the samples without reinforcement, for example with respect to the plane which most often appears parallel to the surface of the sample (i.e., perpendicular to DN), that is, the {101} plane.

**Figure 7:**
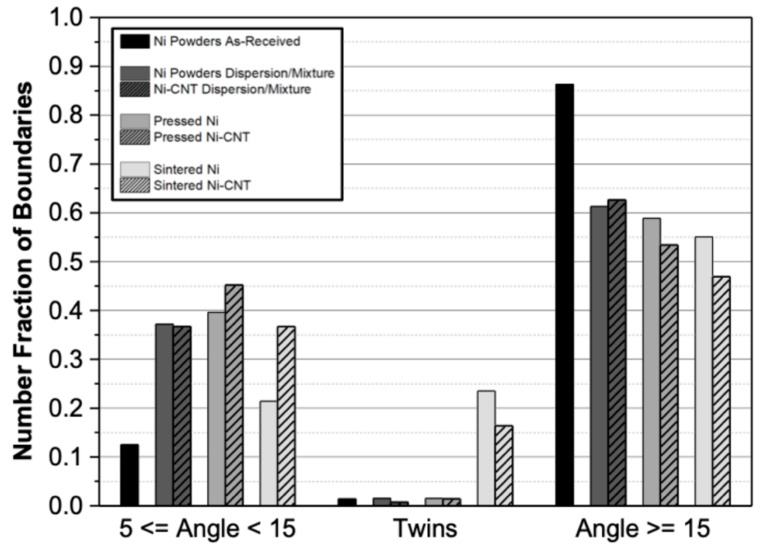
Grain boundary distribution character for the different steps of the powder metallurgy route.

In order to better understand the microstructural changes during the various stages of production, the grain boundary character was studied in more detail. Figure 7 shows the fraction of low- and high-angle boundaries as well as twin boundaries for the samples with and without CNTs, after different production steps. It is important to mention that the distribution of boundary character was performed based on EBSD analysis, so the values are influenced by the specific area analysed and should be used only as a reference in comparing the different samples.

The results presented in Figure 7 revealed that the as-received Ni powders (black columns on the chart) are mainly composed of high-angle boundaries, showing a fraction of less than 0.2 of low-angle boundaries located near the particle surfaces (as observed in Figure 2c,d). Twin boundaries were almost absent on Ni powders.

The dispersion/mixing and pressing processes induce the formation of low-angle boundaries for both samples. That is explained by the increase in dislocation density associated with the plastic deformation during the ultrasonication and pressing steps.

For the sintered samples, the effect of the CNTs on the microstructure of the nanocomposites consisted of a significant decrease in the fraction of the low-angle boundaries observed for the sintered Ni sample when compared to those present in the samples after pressing. Ni-CNT nanocomposites did not show the same behaviour; only a slight decrease in low-angle boundaries was observed, which reveals the influence of CNTs in recovery, recrystallization and grain growth during the sintering process. A noticeable increase in the fraction of twin boundaries was observed for both samples in comparison with other production stages, being more significant for the samples without reinforcement. This effect can be associated with recrystallization, since the formation of twins (Σ3 and Σ9) is accompanied by an elimination of the dislocation structure.

To sum up, the presence of CNTs has a significant effect on the microstructure of the nanocomposite only during the sintering step, in which the reinforcement affects recrystallization. For the Ni sample, due to the plastic deformation induced by the previous processes, the densification of the powders is accompanied by recrystallization and some grain growth. For Ni-CNT nanocomposites, recovery, recrystallization and grain growth occurred to a smaller extent. The CNTs act as obstacles to dislocation annihilation and grain boundary migration, which hinders the elimination of plastic deformation during the sintering process.

## 4. Conclusions

In this research, the effect of CNTs on the microstructure of the Ni-CNT nanocomposites produced by powder metallurgy was evaluated. After the initial dispersion/mixing and pressing processes, the microstructure of the samples with and without CNTs is similar. Both samples are characterized by an increase in the low-angle boundaries fraction and grain misorientation that are related to the plastic deformation that occurs in these steps. Dislocation microstructures formed in both samples evolve into dislocation cells. The accumulation of dislocations on cell boundaries led to grain boundaries inducing smaller grain size in localized regions. The effect of CNTs on the microstructure of nanocomposites is evident only after sintering. While for the samples without CNTs the sintering promoted the almost complete elimination of the dislocation structure, for the nanocomposites this process did not occur to the same extent. The sintered Ni samples are characterized by recrystallized grains and a small fraction of low-angle boundaries. For the nanocomposites, the fraction of low-angle boundaries is significantly higher, and a dislocation structure is still observed. The CNTs have a significant effect on the microstructure of the nanocomposites, since they inhibit the mobility of the grain boundaries and the rearrangement of the dislocations affecting the recovery and recrystallization processes.
